# The association of dietary phytochemical index and nonalcoholic fatty liver disease

**DOI:** 10.1002/fsn3.3389

**Published:** 2023-05-03

**Authors:** Bijan Ahmadi, Amirhossein Ramezani Ahmadi, Mohamadreza Jafari, Nava Morshedzadeh

**Affiliations:** ^1^ Gastroenterology and Hepatology Research Center Kerman University of Medical Sciences Kerman Iran; ^2^ Isfahan Endocrine and Metabolism Research Center Isfahan University of Medical Sciences Isfahan Iran; ^3^ Department of Nutrition, Faculty of Public Health Kerman University of Medical Sciences Kerman Iran

**Keywords:** dietary phytochemical index, liver enzymes, metabolic parameters, nonalcoholic fatty liver disease

## Abstract

Consumption of phytochemical‐rich foods relates to the prevention of chronic diseases. In this study we assessed the dietary phytochemical index (PI) in metabolic parameters, liver enzymes, and severity of fibrosis among nonalcoholic fatty liver disease patients. This cross‐sectional study was conducted on 210 patients with NAFLD. Fibrosis‐4 index (FLB4), nonalcoholic fatty liver disease fibrosis score (NFS), FBS, lipids profile, AST, ALT, ALP, and GGT were measured. PI was calculated through the information obtained from a validated semi‐quantitative food frequency. Multiple regression models were used to estimate mean difference changes in the evaluated variables associated with various dietary PI. Participants' mean ± SD of age and BMI were 39.23 ± 10.52 and 24.40 ± 2.64, respectively. We found that DPI is inversely associated with serum TG, TC, and LDL‐C and directly associated with serum HDL‐C and a higher score in DPI is associated with lower scores in NFS and FIB‐4. Multivariate linear regression showed that there is an inverse association between DPI and AST, ALT, ALP, GGT, NFS, and FIB‐4. Higher dietary PI could impact on reduction of NAFLD progression and improvement of metabolic parameters.

## Graphical abstract

This article evaluates the relationship between dietary intake of phytochemicals and indicators of nonalcoholic fatty liver disease from different aspects.

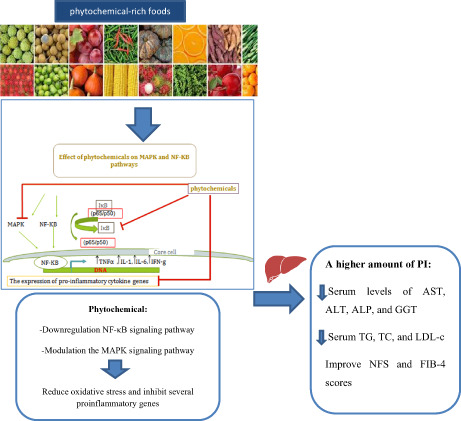

## 1 INTRODUCTION

Nonalcoholic fatty liver disease (NAFLD) is the important reason behind chronic liver disease worldwide (Jarvis et al., 2020). NAFLD is a metabolic dysfunction condition characterized by microvascular steatosis in ≥5% of hepatocytes in people consuming little or no alcohol (<10 gr) (Jarvis et al., 2020; Loomba & Sanyal, 2013). NAFLD ranges from hepatic steatosis without inflammation to steatohepatitis, which can cause fibrosis, cirrhosis, and cancer (Lazarus et al., 2022). Approximately the condition of one of five patients with NAFLD terminates in nonalcoholic steatohepatitis (NASH) (Lazarus et al., 2022). Prevalence of NAFLD in Iran has been reported to be 2.9%–7.1% in the general population (Adibi et al., 2017). NASH usually occurs more in woman than in men, obese Hispanic, adults with body mass index (BMI) > 40 (morbid obesity), and older people especially in people with sarcopenia (Adams et al., 2005; Dreher, 2018). NAFLD may affect all age groups, with middle‐aged woman appearing to be major group of patients with NAFLD (Loomba & Sanyal, 2013). NAFLD can have a cause and effect relationship with metabolic syndrome and its considered as a multifactorial disease (Lazarus et al., 2022). Study shows strong associations between the degree of obesity and prevalence as well as severity of NAFLD (Adams et al., 2005). Prevalence of NAFLD is higher in patients with type 2 diabetes and reciprocally incidence of T2DM is higher in patients with NAFLD (Lazarus et al., 2022).

A meta‐analysis conducted in 2020 indicated that lipid metabolism abnormalities such as high triglycerides, low high‐density lipoprotein (HDL), and hypercholesterolemia with hypertension and metabolic syndrome were associated with prognosis of incidence of severe liver damage in patients with NAFLD (Jarvis et al., 2020). Furthermore, it has been shown that PNPLA3 (encoding patatin‐like phospholipase domain‐containing protein 3) and TM6SF2 (encoding transmembrane 6 superfamily member 2) genes are related to NAFLD (Younossi et al., 2018). In addition to them, environmental factors have a main role in NAFLD risk. The most important environmental factors include dietary pattern, physical activity, and socioeconomic factors. Changes in dietary habits such as increased consumption of carbohydrates, processed meats, added sugars, which play an important role in the progression of NAFLD, result in increased prevalence of this chronic disease (Juanola et al., 2021).

Phytochemicals are secondary (subsidiary) metabolites of plants which may exert many biological activities in humans (Molyneux et al., 2007). These nonnutritive substances are present in fruits, vegetables, whole grain, seeds, legumes, and other food plants (Bahadoran et al., 2015). Phytochemicals are classified into different group such as alkaloids, phenolics, carotenoids, terpenes, saponins, glucosinolates, cyanogenic glycosides, each showing a different biological activity (Krzyzanowska et al., 2010).

Recent studies show that higher consumption of phytochemical‐rich foods is related to reduce hyperinsulinemia and insulin resistance (Bahadoran et al., 2015). Some studies indicate that catechin, as a major flavonoid in green tea, could ameliorate insulin resistance in patients with NAFLD (Sakata et al., 2013). Green, black, and dark teas could moderate NAFLD by improving liver function, lowering the levels of reactive oxygen species (ROS) and inflammatory cytokines, as well as regulating glucose and lipid metabolism (Li et al., 2021). Resveratrol is a one of subclasses of polyphenols found in grapes, red wines, peanuts, and berries. It has been revealed that consuming resveratrol in patients could reduce the aspartate transaminase (AST), alanine transaminase (ALT), fasting blood sugar, fasting blood sugar, and insulin resistance compared to controlled group (Bagherniya et al., 2018). Anthocyanin extracted from sweet cherry has been shown to affect NAFLD. ACNs reduce hepatocellular lipid accumulation by inhibiting lipogenesis and promoting lipolysis (Jia et al., 2013).

Carotenoids are other plant bioactive compounds found in brightly colored vegetables and fruits. Human studies show that consuming carotenoid derivatives can improve NASH and NAFLD (Lee et al., 2019). In short, antioxidant compounds such as flavononols resveratrol, anthocyanins, lycopene, quercetin could be effective factors for alleviating NAFLD because of their significant impacts on modulation lipid metabolism, inflammatory response, oxidative stress by regulating AMP‐activated protein kinase (AMPK), peroxisome proliferator‐activated receptors α/γ (PPARα/γ), nuclear factor erythroid 2‐related factor 2 (Nrf2), and mammalian target of rapamycin (mTOR) signaling pathways (Bagherniya et al., 2018).

However, to our knowledge, no study has been conducted to explore the association between dietary phytochemical index (DPI)‐ and NAFLD‐related parameters in Iranian adults. Thus, the current study aimed to analyze the relationship between DPI and NAFLD.

## 2 MATERIALS AND METHODS

### 2.1 Study population

This cross‐sectional study was conducted on 210 patients with NAFLD in Kerman city, Iran in 2020. The criteria for diagnosing NAFLD by gastroenterologists were based on histopathological and laboratory results, to assess for fibrosis: Fibrosis‐4 index (FLB4) and nonalcoholic fatty liver disease fibrosis score (NFS). Patients entered the study and allocated to two groups according to clinical diagnosis by random sampling method. The other inclusion criteria were: no consumption of alcohol over the previous 3 months; the absence of other liver disorders, cancer, CVD, respiratory and renal disorders, diabetes, no history of bariatric surgery in recent years, no drug consumption over the prior 3 months, and irreversible endocrine. Exclusion criteria were pregnancy or breastfeeding, reduced average body weight as >5% over the past 6 months or in low‐calorie diet and unwillingness to continue the participation.

### 2.2 Anthropometric assessment and physical activity

Weight was taken to the nearest 100 g, using digital scales with the individuals wearing minimal clothing and no shoes. Height was recorded to the nearest 0.1 cm using in a standing position without shoes. Body mass index was calculated as weight (kg) divided by square of the height (m^2^). Blood pressure was determined after 10 min rest and in a sitting position blood pressure was measured twice on the right arm using a standard mercury sphygmomanometer (Riester).

Physical activity data were assessed via a valid self‐reported questionnaire and calculated as metabolic equivalent hours per day (MET.h.d), which has been evaluated and validated for the population of Iran (Aadahl & Jørgensen, 2003).

### 2.3 Biochemical measurements

Fasting blood glucose level was determined by the enzymatic colorimetric method using glucose oxidase. Enzymatic total cholesterol (TC) was measured with cholesterol esterase and cholesterol oxidase using an enzymatic colorimetric method. Serum levels HDL‐C and serum triglyceride (TG) were assayed using an enzymatic colorimetric method. Low‐density lipoprotein cholesterol (LDL‐C) was determined via Friedewald formula. Albumin and platelet count were evaluated using an automated hematology analyzer.

A colorimetric coupled enzyme assay for the determination of gamma‐glutamyltransferase (GGT) activity, AST, and alkaline phosphatase (Parsazmoun).

FIB‐4 and NFS were developed to assess the severity of hepatic fibrosis noninvasively. FIB‐4 and NFS are the two important noninvasive indexes for screening advanced fibrosis individuals with NAFLD. FIB‐4 and NFS indicate elevated liver stiffness as an indicator of hepatic fibrosis.

The FIB4‐index was calculated based on six variables, including age, serum AST and ALT levels, BMI impaired fasting glucose (IFG), and platelet counts [1.625 + 0.037 × age (years) + 0.094 × BMI (kg/m^2^) + 1.13 × IFG/diabetes mellitus (DM) (yes = 1, no = 0) + 0.99 × aspartate aminotransferase (AST): alanine aminotransferase (ALT) ratio − 0.013 × platelet counts (×10^9^/L) – 0.66 × albumin (Alb) (g/dL)] (Ledikwe et al., 2004; Mirmiran et al., 2012). NFS was calculated using the following formula: −1.675 + 0.037 × age (year) + 0.094 × BMI (kg/m^2^) + 1.13 × IFG/diabetes (yes = 1, no = 0) + 0.99 × AST/ALT ratio – 0.013 × platelet count (×10^9^/L) – 0.66 × albumin (g/dL).

### 2.4 Calculation of phytochemical index

Dietary data were assessed using a validated semiquantitative food frequency questionnaire (FFQ) with 168 food items. Typical dietary intakes of individuals over the past year were tested using a valid and reliable semiquantitative FFQ (Asghari et al., 2012; Rezazadeh et al., 2020). The FFQ consisted of 148 food items with standard portion sizes commonly consumed by this population. The participants were asked to illustrate their usual intake frequency of over the past year on a daily, weekly, or monthly basis. All reported intake frequencies were converted into grams per day using standard measures. Then, mean daily intakes of micro and macronutrients for each individual were determined using the United States Department of Agriculture (USDA) food composition table.

The DPI calculation method developed by McCarty (2004) is based on this formula: [DPI = (daily energy obtained from foods rich in phytochemicals (kcal)/total daily energy intake (kcal)) × 100] (McCarty, 2004). Fruits, vegetables (natural fruit and vegetable juices), legumes, whole grains, nuts, soy products, seeds, and olive oil were considered as phytochemical‐rich foods (Mofrad et al., 2019).

### 2.5 Statistical methods

To compare study variables, participants were categorized into the quartiles according to the DPI score. Continuous and categorical variables were reported as mean ± SD and frequency (%), respectively. The normality of continuous variables was evaluated using the Shapiro–Wilk test. To compare differences of continuous variables across the DPI quartiles, the one‐way analysis of variance (ANOVA) with a Bonferroni post‐hoc test was used. Categorical variables were compared across the DPI quartiles using the Chi‐square or the Fisher's exact tests. In the multivariate linear regression, the association between continuous dependent variables and quartiles of DPI (as an independent variable) was evaluated in crude or adjusted model. In adjusted model, the associations were adjusted according to gender, BMI, physical activity, and energy intake. The SPSS software (Version 26.0; Chicago, IL) was used to perform statistical analyses. The *p*‐values < .05 were considered statistically significant.

## 3 RESULTS

### 3.1 General characteristics

Overall, 200 patients with NAFLD were analyzed in the present study. Participants' mean ± SD of age and BMI were 39.23 ± 10.52 and 24.40 ± 2.64, respectively. Fifty percent (*n* = 100) of the participants were men. The mean ± SD of NFS (min = −1.62, max = 0.90) and FBS (min = 1.30, max = 3.90) were 0.57 ± 0.54 and 2.89 ± 0.69, respectively. The DPI ranged between 15.50 and 38.80. The general characteristics across the quartiles of the DPI are presented in Table 1. The height, weight, BMI, physical activity, gender, and medication use were significantly different across the quartiles of DPI (*p* < .001). However, no association was observed between DPI score and age, education, marital status, smoking, alcohol consumption, ethnicity, or the presence of other chronic diseases (*p* < .05). The association between the dietary intake and DPI is reported in Table 2. The DPI had no significant association with the intake of energy, carbohydrates, protein, fatty acids, and fiber (*p* > .05).

**TABLE 1:** General characteristics of participants according to the dietary phytochemical index (DPI) quartiles (*n* = 200).

DPI		Total		Q1		Q2		Q3		Q4		*p*‐Value^1^
		Mean or *N*	SD or %	Mean or *N*	SD or %	Mean or *N*	SD or %	Mean or *N*	SD or %	Mean or *N*	SD or %	
Age (years)		39.23	10.52	37.84	10.92	39.01	9.80	41.59	10.75	38.51	10.55	.311
Height (cm)		167.27	5.88	172.39^a^	6.89	166.79^a,b^	3.78	166.3^a,c^	5.46	163.53^a,b,c^	2.70	**<.001**
Weight (kg)		68.43	9.37	81.18^a^	6.52	69.35^a,b^	3.06	64.58^a,b,c^	3.52	58.29^a,b,c^	2.86	**<.001**
BMI (kg/m^2^)		24.40	2.64	27.38^a^	2.40	24.94^a,b^	1.23	23.40^a,b,c^	1.68	21.80^a,b,c^	1.07	**<.001**
Physical activity (METs/week)		420.82	196.36	248.64^a^	34.31	322.65^a,b^	60.83	430.85^a,b,c^	114.58	690.67^a,b,c^	160.74	**<.001**
Gender	Male	100	50	41	82	20	38.5	18	36.7	21	42.9	**<.001**
	Female	100	50	9	18	32	61.5	31	63.5	28	57.1	
Education	Diploma and less	34	17	9	18	4	7.7	9	18.4	12	24.5	.293
	Bachelor and master	98	49	28	56	26	50	23	46.9	21	42.9	
	Ph.D. and above	68	34	13	26	22	42.3	17	34.7	16	32.7	
Marital status	Married	144	72	37	74	37	71.2	40	81.6	30	61.2	.163
	Single	56	28	13	26	15	28.8	9	18.4	19	38.8	
Smoking	Active	47	23.5	15	30	11	21.2	7	14.3	14	28.6	.099
	Passive	44	22	13	26	6	11.5	12	24.5	13	26.5	
	No	109	54.5	22	44	35	67.3	30	61.2	22	44.9	
Alcohol use	Yes	18	9	9	18	3	5.8	2	4.1	4	8.2	.100
	No	182	91	41	82	49	94.2	47	95.9	45	91.8	
Other chronic diseases	Yes	20	10	4	8	7	13.5	6	12.2	3	6.1	.590
	No	180	90	46	92	45	86.5	43	87.8	46	93.9	
Medication use for NAFLD	Yes	142	71	46	92	38	73.1	34	69.4	24	49	**<.001**
	No	58	29	4	8	14	26.9	15	30.6	25	51	
Ethnicity	Fars	115	57.5	28	56	31	59.6	28	57.1	28	57.1	.986
	Other	85	42.5	22	44	21	40.4	21	42.9	21	42.9	

*Note*: ^a,b,c,d^Similar letters have a significant difference (*p* < .05); Bonferroni post‐hoc test was used to calculate *p*‐value.

Abbreviations: DPI, dietary phytochemical index; SD, standard deviation.

^1^ Calculated using one‐way ANOVA for quantitative variables and Chi‐square for qualitative variables. In these tests, bold values indicatethe significance level below 0.05 is defined (*p* < .05).

**TABLE 2:** Dietary intakes according to the dietary phytochemical index (DPI) quartiles (*n* = 200).

DPI	Q1		Q2		Q3		Q4		*p*‐Value^1^
	Mean	SD	Mean	SD	Mean	SD	Mean	SD	
Energy (kcal/day)	2057.55	400.97	2085.26	273.80	2073.77	310.98	2091.45	210.53	.950
Carbohydrate (g/day)	312.72	68.74	318.20	49.64	313.16	55.79	305.35	60.01	.773
Protein (g/day)	73.58	16.77	72.96	11.72	68.24	10.54	70.31	14.92	.200
Total fat (g/day)	58.44	13.09	59.49	11.58	58.45	15.64	56.43	12.84	.739
Cholesterol (mg/day)	165.66	53.96	178.48	49.54	165.67	49.13	171.78	47.10	.530
SFAs (g/day)	18.06	4.96	18.36	5.11	17.06	4.68	16.87	4.46	.351
MUFAs (g/day)	21.29	4.89	21.68	4.02	21.42	5.31	20.84	4.69	.861
PUFAs (g/day)	12.53	3.18	12.57	2.37	12.92	2.53	12.31	3.18	.780
ω‐3 FAs (g/day)	10.52	2.48	10.67	2.23	11.05	2.31	10.43	2.62	.618
ω‐6 FAs (g/day)	0.05	0.04	0.04	0.03	0.03	0.02	0.04	0.02	.193
Total fiber (g/day)	31.14	12.52	31.94	10.90	31.77	10.74	27.86	9.42	.263

Abbreviations: MUFAs, mono‐unsaturated fatty acids; PUFA, polyunsaturated fatty acids; SD, standard deviations; SFAs, Saturated fatty acids.

^1^ Calculated using one‐way ANOVA. In this test, the significance level below 0.05 is defined (*p* < .05).

### 3.2 Lipid profile

The association between DPI and the lipid profile is outlined in Tables 3 and 4. The multivariate linear regression revealed that DPI is inversely associated with serum TG, TC, and LDL‐C (*p*‐trend < .001). At the other hand, a direct association was observed between the DPI and serum HDL‐C (*p*‐trend < 0.001). The significance of these associations remained unchanged after adjusting for the effects of gender, BMI, physical activity, and energy intake.

**TABLE 3:** Lipid profile and severity of NAFLD according to the dietary phytochemical index (DPI) quartiles (*n* = 200).

DPI		Q1		Q2		Q3		Q4		*p*‐Value^1^
		Mean or *N*	SD or %	Mean or *N*	SD or %	Mean or *N*	SD or %	Mean or *N*	SD or %	
TG (mg/dL)		273.50^a^	28.96	240.95^a,b^	37.61	212.92^a,b,c^	28.37	190.52^a,b,c^	21.07	**<.001**
TC (mg/dL)		233.94^a^	19.82	218.31^a,b^	12.91	206.90^a,b,c^	14.18	197.25^a,b,c^	13.12	**<.001**
LDL‐C (mg/dL)		159.95^a^	20.95	140.02 ^a,b^	23.67	120.32^a,b,c^	22.79	106.54^a,b,c^	16.28	**<.001**
HDL‐C (mg/dL)		37.85^a^	3.46	39.43^b^	3.04	40.85^a^	2.99	42.22^a,b^	3.29	**<.001**
AST (IU/L)		76.31^a^	8.12	64.50^a,b^	6.89	58.74^a,b,c^	4.82	52.62^a,b,c^	3.59	**<.001**
ALT (IU/L)		79.44^a^	7.57	68.68^a,b^	6.55	62.67^a,b,c^	4.81	57.20^a,b,c^	3.39	**<.001**
ALP (IU/L)		195.94^a^	21.75	186.50^a,b^	14.53	181.88^a^	12.49	173.83^a,b^	10.25	**<.001**
GGT (IU/L)		102.69^a^	18.80	92.63^a,b^	15.70	89.76^a,c^	16.57	77.57^a,b,c^	9.53	**<.001**
NFS		0.77^a^	0.08	0.63^b^	0.47	0.61^c^	0.44	0.24^a,b,c^	0.79	**<.001**
FIB‐4		3.47^a^	0.36	3.10^a,b^	0.57	2.69 ^a,b,c^	0.60	2.29^a,b,c^	0.60	**<.001**
Grade of NAFLD (%)	I	0	0	2	3.8	12	24.5	45	91.8	**<.001**
	II	5	10	31	59.6	33	67.3	4	8.2	
	III	45	90	19	36.5	4	8.2	0	0	

*Note*: ^a,b,c,d^Similar letters have a significant difference (*p* < .05); Bonferroni post‐hoc test was used to calculate *p*‐value.

Abbreviations: ALP, alkaline phosphatase; ALT, alanine aminotransferase; AST, aspartate aminotransferase; FIB‐4, Fibrosis‐4; GGT, gamma‐glutamyl transferase; HDL‐C, high‐density lipoprotein cholesterol; LDL‐C, low‐density lipoprotein cholesterol; NAFLD, nonalcoholic fatty liver disease; NFS, NAFLD fibrosis score; SD, standard deviation; TC, total cholesterol; TG, triglyceride.

^1^ Calculated using one‐way ANOVA for quantitative variables and Fisher's exact test for qualitative variable. In these tests, bold values indicatethe significance level below 0.05 is defined (*p* < .05).

**TABLE 4:** Multivariate linear regression of the association between lipid profile, severity of NAFLD, and dietary phytochemical index (DPI) (*n* = 200).

Variable	Model	DPI								*p*‐Trend
		Q1		Q2		Q3		Q4		
		*β* (SE)	*p*	*β* (SE)	*p*	*β* (SE)	*p*	*β* (SE)	*p*	
TG (mg/dL)	Crude^1^	Ref.	–	−32.55 (5.82)	**<.001**	−60.57 (5.91)	**<.001**	−82.97 (5.91)	**<.001**	**<.001**
	Adjusted^2^	Ref.	–	−36.13 (6.81)	**<.001**	−65.15 (8.13)	**<.001**	−86.31 (11.39)	**<.001**	**<.001**
TC (mg/dL)	Crude	Ref.	–	−15.63 (2.99)	**<.001**	−27.04 (3.03)	**<.001**	−36.69 (3.03)	**<.001**	**<.001**
	Adjusted	Ref.	–	−13.70 (3.51)	**<.001**	−24.60 (4.19)	**<.001**	−34.23 (5.87)	**<.001**	**<.001**
LDL‐C (mg/dL)	Crude	Ref.	–	−19.93 (4.14)	**<.001**	−39.63 (4.21)	**<.001**	−53.41 (4.21)	**<.001**	**<.001**
	Adjusted	Ref.	–	−19.82 (4.88)	**<.001**	−39.48 (5.83)	**<.001**	−52.51 (8.17)	**<.001**	**<.001**
HDL‐C (mg/dL)	Crude	Ref.	–	1.57 (0.62)	**.012**	2.99 (0.63)	**<.001**	4.36 (0.63)	**<.001**	**<.001**
	Adjusted	Ref.	–	1.32 (0.73)	.072	2.51 (0.88)	**.004**	3.61 (1.23)	**.003**	**.002**
AST (IU/L)	Crude	Ref.	–	−10.12 (1.34)	**<.001**	−13.96 (1.61)	**<.001**	−16.22 (2.25)	**<.001**	**<.001**
	Adjusted	Ref.	–	−11.80 (1.20)	**<.001**	−17.56 (1.22)	**<.001**	−23.69 (1.22)	**<.001**	**<.001**
ALT (IU/L)	Crude	Ref.	–	−10.75 (1.14)	**<.001**	−16.76 (1.16)	**<.001**	−22.23 (1.16)	**<.001**	**<.001**
	Adjusted	Ref.	–	−8.87 (1.27)	**<.001**	−12.99 (1.52)	**<.001**	−14.91 (2.13)	**<.001**	**<.001**
ALP (IU/L)	Crude	Ref.	–	−9.44 (3.02)	**.002**	−14.06 (3.06)	**<.001**	−22.10 (3.06)	**<.001**	**<.001**
	Adjusted	Ref.	–	−0.12 (3.28)	.969	1.49 (3.92)	.703	3.67 (5.49)	.504	.518
GGT (IU/L)	Crude	Ref.	–	−10.05 (3.05)	**.001**	−12.92 (3.09)	**<.001**	−25.11 (3.09)	**<.001**	**<.001**
	Adjusted	Ref.	–	−5.51 (3.54)	.120	−5.77 (4.22)	.172	−14.78 (5.92)	**.013**	**.041**
NFS	Crude	Ref.	–	−0.13 (0.10)	.173	−0.16 (0.10)	.108	−0.53 (0.10)	**<.001**	**<.001**
	Adjusted	Ref.	–	−0.05 (0.11)	.619	−0.05 (0.13)	.690	−0.36 (0.19)	.061	**.179**
FIB‐4	Crude	Ref.	–	−0.37 (0.10)	**.001**	−0.778 (0.10)	**<.001**	−1.18 (0.10)	**<.001**	**<.001**
	Adjusted	Ref.	–	−0.40 (0.12)	**.001**	−0.88 (0.14)	**<.001**	−1.38 (0.20)	**<.001**	**<.001**

Abbreviations: ALP, alkaline phosphatase; ALT, alanine aminotransferase; AST, aspartate aminotransferase; FIB‐4, Fibrosis‐4; GGT, gamma‐glutamyl transferase; HDL‐C, high‐density lipoprotein cholesterol; LDL‐C, low‐density lipoprotein cholesterol; NAFLD, nonalcoholic fatty liver disease; NFS, NAFLD fibrosis score; SD, standard deviation; TC, total cholesterol; TG, triglyceride.

^1^ Calculated using multivariate linear regression, DPI quartiles entered as an independent variable.In this test, bold values indicate the significance level below 0.05 is defined (*p* < .05).

^2^ Calculated using multivariate linear regression, DPI quartiles entered as an independent variable, and adjusted for the effect of gender, BMI, physical activity, and energy intake.

### 3.3 Liver enzymes and severity of NAFLD

Table 3 shows that a higher score in DPI (fourth quartile) is associated with a lower serum level of AST, ALT, ALP, and GGT compared to the first quartile (*p* < .001). Moreover, it was found that a higher score in DPI is associated with lower scores in NFS and FIB‐4 (*p* < .001). Also, worse results in sonographic assessment of liver were associated with a lower DPI score (*p* < .001). Multivariate linear regression showed that the DPI is inversely associated with AST, ALT, ALP, GGT, NFS, and FIB‐4 (*p*‐trend < .001). However, the ALP and NFS were not associated with DPI after adjusting for the effect of gender, BMI, physical activity, and energy intake (*p*‐trend = .518 and .179, respectively).

## 4 DISCUSSION

It has been accepted by scientists that NAFLD is one of the most progressive liver diseases around the world. Among the many factors affecting NAFLD, lifestyle related and dietary habits are the momentous modifiable factors in NAFLD suppression and treatment (Bahadoran et al., 2015). Plant‐based foods have a high fiber and lower energy substance than processed food and rich in phytochemicals (Asgari et al., 2021). Recent researches have shown that phytochemicals had some impacts on NAFLD.

This study evaluated association between PI and severity of NAFLD as well as metabolic paraments in patients with NAFLD. It was found that a higher score in DPI is related to lower scores in NFS and FIB‐4. A higher amount of PI was inversely related to the serum levels of AST, ALT, ALP, and GGT.

Phytochemicals can improve lipid profile (Carter et al., 2010). The results of recent studies and clinical trials that have evaluated the relation between phytochemical‐rich plant food consumption and serum concentrations of TG, TC, and LDL‐C reached a similar result regarding the improvement of lipid profile (Giglio et al., 2016; Golzarand et al., 2014; Toth et al., 2016). A phytochemical‐rich diet can be found in various components of healthy eating habits, such as the Mediterranean diet or Dietary Approaches to Stop Hypertension (DASH) diet. Some studies have shown Mediterranean diet adherence was inversely associated with LDL‐C and TC/HDL‐C ratio, while positively linked with HDL‐C. Furthermore, consumption of fruits and nuts was significantly inversely associated with TC, LDL‐C, and TC/HDL‐C ratio (Fitó et al., 2007; Mertens et al., 2014).

Delshad Aghdam et al. (2021) reported higher intake of foods which are rich in phytochemicals was associated with lower FBG, LDL‐C, as well as a simultaneous increase in HDL‐C levels. Nevertheless, other studies have not found any obvious relationship between FBG level and DPI (Bahadoran et al., 2012, 2013).

It has been made clear that intake of fiber with very rich in insoluble polyphenols has beneficial effects on the LDL‐C/HDL‐C ratio (Ruiz‐Roso et al., 2010). However, in another study observed no relationship between HDL‐C level and DPI in healthy individuals (Bahadoran et al., 2012). Numerus studies have demonstrated the advantageous effects of increasing consumption of foods rich in phytochemical in the diet such as Mediterranean diet and high nutrient dietary patterns (i.e., fruits, nuts, olive oil, dark green/yellow vegetables, olive oil) on waist circumference (WC) and BMI (He et al., 2004; Ledikwe et al., 2004).

A previous study reported a significantly reverse relationship between the highest quartile categories of dietary PI and weight as well as WC. It was found that a higher dietary PI score in DPI was linked with 1.47% reduction in BAI in 3 years of follow‐up (Mirmiran et al., 2012).

A cross‐sectional study reported that PI score was conversely related to adiposity, BMI, WC, and oxidative stress in healthy young adults (Vincent et al., 2010). Another cross‐sectional study found that children in the upper quartile of phytochemicals consumption had significantly lower chance of being overweight/obese compared to those in the first quartile of phytochemical consumption (Eslami et al., 2020). Natural or pharmacological substances (fatty acids, fiber, and polyphenols) predominately regulate the expression of genes involved in lipid metabolism. Omega‐3 polyunsaturated fatty acids (PUFAs) and their metabolites are the ligands that found in nature for peroxisome proliferator receptor activator (PPARγ) (Poirier et al., 2001). PPARγ expression is mostly occur in tissues such as liver, adipose tissue where it induces the β‐oxidative degradation of fatty acids (Grygiel‐Górniak, 2014). Peroxisome proliferator response elements (PPREs) regulate the gene expression by associating with activator proteins independently of PPREs, which modulate the genes contributing to the release, transport, as well as storage of fatty acids such as lipoprotein lipase (LPL) (Chinetti et al., 2000).

Our study revealed that high DPI score is associated with lower means of AST, ALT, ALP, and GGT as well as NFS and FIB‐4 scores as markers of severity of NAFLD. Limited studies have been done in the field of dietary phytochemical index and NAFLD.

Recent studies have shown that consuming phytochemicals is associated with lowered levels of insulin, lipid profiles, ALT, AST, and GGT (Francini‐Pesenti et al., 2019). On the contrary, a cross‐sectional study indicated converse association between DPI score and serum ALP, though no notable correlation was found between DPI score and serum levels of AST, ALT, as well as GGT in healthy participants (Darabi et al., 2022). The mechanisms involved in steatosis–NASH transition are multifactorial and have not been completely determined. Nevertheless, characteristically, fat accumulation‐driven hepatic inflammation are key actors in NAFLD progression (Gao & Tsukamoto, 2016).

There are numerus mechanisms that could describe the effects of phytochemical‐rich diets against NAFLD. Plant‐derived bioactive compounds can affect many inflammatory processes. A wide spectrum of phytochemicals with different chemical structures have proved to be associated with various anti‐inflammatory mechanistic effects (Zhu et al., 2018). Flavonoids and flavones from fruits inhibit a range of proinflammatory inductor, such as those derived from the arachidonic acid pathway and nuclear factor kappa B (NF‐κB) activation, downregulating tumor necrosis factor alpha (TNF‐α), interleukin 1 beta (IL‐1β) expression and secretion as well as vascular cell adhesion molecule‐1 (VCAM‐1) expression (Vafeiadou et al., 2009; Wang et al., 2008).

Zhang et al. (2015) explain that supplementation with anthocyanins for 12 weeks was significantly converse correlation with the level of ALT and improved the fibrosis scores of patients with NAFLD. Additionally, based on the results of a meta‐analysis, anthocyanins consumption had no specific impact on GGT, AST, and ALT levels. Although, it seems that anthocyanins can effect on affect AST levels, among healthy individuals that used ACNs‐rich products rich as intervention (Sangsefidi et al., 2021). Contradictory results from different studies due to differences in study design, sample size, and the participants' genetic characteristics. The results from some studies suggest that the mTOR inhibitory effect of anthocyanins through the activation of AMPK causes modulation of lipid metabolism and fat deposition in the liver (Lee et al., 2010; Vendrame et al., 2014). ACNs also decrease oxidative stress by promoting the antioxidant response by downregulating phosphorylation of mTOR and Akt (Lee et al., 2010).

Anthocyanins interfere with inflammatory processes via downregulation of the redox‐sensitive nuclear factor‐κB signaling pathway and modulation of the MAPK signaling pathway to reduce oxidative stress and inhibit several proinflammatory genes (Behl et al., 2022; Vendrame & Klimis‐Zacas, 2015). Flavanones mediate anti‐inflammatory effects by preventing the phosphorylation of extracellular signal regulated kinases (ERK)1/2 and activity of p38 mitogen‐activated protein kinase (MAPK) (Chen et al., 2016). Furthermore, some studies have shown that genistein, as a phytoestrogen, suppressed activations of signal transducer and activator of transcription 1 (STAT1) and NF‐κB p65 pathway (Hämäläinen et al., 2007; Kim & Kim, 2011).

The anti‐inflammatory effect of phytochemical compounds has been identified to impact Toll‐like receptor 4 (TLR4) signaling pathways. Evidence indicates that phytochemicals targeting TLR4 are potentially impressive for a new treatment strategy for patients with IBD (Dai et al., 2022).

Zeng et al. (2013) used experimental models to examine the anti‐inflammatory potential of curcumin in the inflammatory colon model. It has been shown that treatment with curcumin can decrease the levels of TLR4 and NF‐κB as well as myeloperoxidase (MPO) activity.

Furthermore, curcumin could ameliorate NAFLD by lowering the levels of lipid profiles, AST, ALT inhibiting O‐GlcNAcylation, and decreasing the generation of ROS, plus IFN‐γ in cells (Panahi et al., 2017). Curcumin additionally reduced the steatosis and fibrosis of hepatocytes by modulating/inhibiting the high‐mobility group box 1 (HMGB1)‐NF‐κB translocation to prevent the NASH progression (Afrin et al., 2017).

Some limitations of the current study should be considered. First of all, it has been used food frequency questionnaire for dietary assessment which may lead to inaccurate reporting of usual intake and to misclassification of exposures. Thus, more valid methods such as weighted food records and food composition analysis can be taken into consideration in future research. Although, was tried to mitigate this inaccuracy through completing dietary questionnaires by a trained dietitian. Second, the cross‐sectional design of the study did not allow to determine cause and effect association. To minimize this effect, patients with any underlying acute infection, systemic inflammation diseases were not included in the study. Nevertheless, several confounders including age, gender, BMI, physical activity, and energy intake and education were controlled in this study.

However, notwithstanding these limitations, to our knowledge, this is the first case–control study to analyze the relation between the usual dietary PI and serum levels of AST, ALT, ALP, and GGT, along with NFS and FIB‐4 in patients with NAFLD. Our results can be used to inform the development of strategies and dietary guidelines aimed at reducing NAFLD progression.

## 5 CONCLUSIONS

In conclusion, our investigation suggests that higher adherence to phytochemical‐rich diets may be associated with reduced NAFLD progression. Further studies are required to find the amount of dietary phytochemical consumption and mechanisms of role in liver diseases.

## AUTHOR CONTRIBUTIONS

**Bijan Ahmadi:** Investigation (equal). **Mohamadreza Jafari:** Funding acquisition (equal). **Nava Morshedzadeh:** Investigation (equal); methodology (equal).

## ETHICS STATEMENT

The study protocol was approved by the Ethics Committee of Gastroenterology and Hepatology Research Center at Kerman University of Medical Sciences. (Ethics Number: IR.KMU.AH.REC.1400.108). Patients completed the ethical questionnaire to attend the study. On the other hand, the information received from each patient is reportedly reported without mentioning a person's name.

## Data Availability

All data generated or analyzed during this study are included in this published article.
